# Prevalence and trends of vitamin D deficiency in a Saudi Arabian population: a five-years retrospective study from 2017 to 2021

**DOI:** 10.3389/fpubh.2025.1535980

**Published:** 2025-04-28

**Authors:** Yahya Madkhali, Balamurugan Janakiraman, Faisal Alsubaie, Olayan Albalawi, Saleh Alrashidy, Mohamad Alturki, Mehrunnisha Ahmed, Md Dilshad Manzar, Faizan Kashoo

**Affiliations:** ^1^Department of Medical Laboratory Sciences, College of Applied Medical Sciences, Majmaah University, Al Majma'ah, Saudi Arabia; ^2^SRM College of Physiotherapy, Faculty of Medicine and Health Sciences, SRM Institute of Science and Technology (SRMIST), Chennai, Tamil Nadu, India; ^3^Department of Statistics, Faculty of Science, University of Tabuk, Tabuk, Saudi Arabia; ^4^Medical Laboratory Department, Second Health Cluster, King Khaled Majmaah Hospital, Al Majma'ah, Saudi Arabia; ^5^Department of Nursing, College of Applied Medical Sciences, Majmaah University, Al Majma'ah, Saudi Arabia; ^6^Department of Physical Therapy and Health Rehabilitation, College of Applied Medical Sciences, Majmaah University, Al Majma'ah, Saudi Arabia

**Keywords:** vitamin D deficiency, trends, prevalence, Saudi Arabia, cross sectional analysis

## Abstract

Vitamin D [25(OH)D] deficiency poses a significant global health concern, especially prevalent in developing nations. This retrospective cross-sectional study, conducted at King Khaled Hospital in Majmaah, aimed to investigate the prevalence and trends of vitamin D deficiency among 22,335 individuals from the Saudi population from 2017 to 2021. The population for this study includes new cases visiting hospitals for routine health check-ups or related to various medical conditions, as well as individuals visiting screening camps outside hospitals in remote areas and schools. Patient data, specifically [25(OH)D] concentration measured through blood samples, were assessed by ROCH COBAS e-411 analyzers. The findings revealed a period prevalence of 67.3% (*n* = 15,025) for [25(OH)D] deficiency (<30 ng/mL). This included *n* = 6,274 (28.1%) with insufficient (20–29 ng/mL), *n* = 8,014 (35.9%) with deficient concentration (<20 to 7 ng/mL), and *n* = 737 (3.3%) with severe (<7 ng/mL) [25(OH)D] concentration. Females were predominantly affected, *n* = 10,442 (69.5%), compared to males, *n* = 4,583 (30.5%). The most affected age group was 10–19 years old, with a mean concentration of [25(OH)D] as low as 21.1 ± 11.9 ng/mL. Trend analysis revealed a significant decrease in vitamin D deficiency prevalence from 32% to 9% between 2017 and 2020, with a slight increase to 18% in 2021. The findings of this study necessitate interventions based on age-specific patterns, providing crucial insights for targeted public health strategies aimed at enhancing vitamin D status in the Saudi population, particularly among the most affected groups such as females and younger individuals within the 10-19-year age group.

## 1 Introduction

Vitamin D 25-hydroxy [25(OH)D] deficiency is a widespread and significant public health concern, affecting almost 50% of the global population (1, 2). An optimum vitamin D concentration is a vital component for maintaining bone homeostasis (3). In recent years, there has been a shift in the perception of vitamin D moving beyond its traditional role as a “bone vitamin” to a recognized status as a “multifunctional vitamin.” This transformation stems from reports that highlight the implications of vitamin D, influencing the homeostatic balance of various physiological systems (4). The pleiotropic effects of vitamin D and the rising prevalence of vitamin D deficiency in the general population have garnered substantial scientific attention, fostering comprehensive investigation into the roles and optimal management of vitamin D deficiency (5, 6). Moreover, vitamin D has been associated with a reduced risk of cancer (7), heart disease (8), fractures and falls (9), autoimmune illnesses (10), influenza (11), type-2 diabetes (12), and depression (13). In a prospective cohort study by McCarthy et al. (14), data from The Irish Longitudinal Study on Ageing (TILDA) demonstrated a clear association between vitamin D deficiency and glycemic control outcomes. Specifically, individuals with baseline vitamin D concentrations below 30 nmol/L were found to have a 62% increased likelihood of developing prediabetes within 4 years compared to those with vitamin D concentrations above 75 nmol/L (14). Additionally, a deficient vitamin D status was associated with a higher prevalence of diabetes, with a relative risk ratio of 1.5, underscoring the potential of vitamin D status as a modifiable risk factor in the prevention and management of diabetes (14).

A [25(OH)D] insufficiency and deficiency in the Kingdom of Saudi Arabia (KSA) is a well-studied domain in both the clinical and academic sectors (15). Previous studies carried out before 2010 reported a prevalence of vitamin D deficiency ranging from 28 to 81% in Saudi men, women, and adolescents (16). More recent large cross-sectional surveys have also confirmed a widespread prevalence of vitamin D deficiency in the Saudi population (17–21). A systematic review was recently conducted on the effectiveness of vitamin D interventions, including supplementation, dietary modifications, and UVB exposure, among the Saudi population. Findings indicate that oral supplementation is the most effective method, significantly increasing serum vitamin D concentrations, followed by dietary changes and UVB exposure (22). A study conducted in KSA reported seasonal variations in vitamin D concentrations among 1,790 pediatric outpatients, revealing a 69% prevalence of deficiency and insufficiency, with the highest concentrations observed during summer and autumn (23). Another study involving 977 male adolescents found that the interaction between season and vitamin D concentrations significantly influences physical fitness, with the most pronounced effects observed in spring and autumn (24). In the UK-based EPIC-Oxford cohort study, plasma 25-hydroxyvitamin D [25(OH)D] concentrations were measured in 2,107 individuals, revealing that vegetarians had significantly lower concentrations than meat and fish eaters, reflecting typical dietary vitamin D sources. Notably, the disparity in vitamin D concentrations was more pronounced during winter, with up to a 38% difference, compared to a 20% difference in summer, highlighting the influence of sunlight exposure (25). This contrast is particularly significant when compared to dietary habits in KSA, where the population predominantly consumes non-vegetarian foods, potentially influencing higher baseline vitamin D concentrations (26).

While attempts have been made to raise awareness about [25(OH)D] deficiency and its preventive strategies, it is important to continue monitoring trends in the population’s [25(OH)D] status for making informed discussions, and decisions about effective approaches to combat evolving trends in vitamin D insufficiency (27). The updated and recent trends of [25(OH)D] deficiency is important for policymakers and public health experts to ascertain the effectiveness of the current measures of [25(OH)D] deficiency management. There are no studies documenting the recent trends of [25(OH)D] concentration in KSA, especially from 2017 onwards. Since the last decade, the government of Saudi Arabia has implemented public awareness campaigns, school programs, and food fortification with vitamin D. The need for these initiatives is supported by an expert consensus on vitamin D correction, highlighting the widespread deficiency in the region and recommending supplementation for individuals with low levels (28). Therefore, this study aimed to investigate the prevalence of [25(OH)D] deficiency in the Sudair area in KSA over a period of 5 years, spanning from 2017 to 2021.

## 2 Methods

### 2.1 Data collection

The data were collected from January 2017 to December 2021 to examine the prevalence trends of [25(OH)D] deficiency among the civilian population of the Sudair area (25.72°N, 45.70°E) of Riyadh Region, KSA. Sudair district comprises several towns and villages located approximately 150 km north of Riyadh, with Majmaah City being the largest and having the major hospital in the district, King Khaled Hospital. Therefore, all medical records of patients who were tested for [25(OH)D] during the study period were obtained from the King Khaled Hospital. This study was approved by the Ministry of Health in KSA with IRB No: 22-485E in November 2022. All patient data and information were kept secure and confidential. Raw data on [25(OH)D] concentration, as well as patient age and gender, were collected from medical laboratory archives and health records at King Khaled Hospital in Majmaah, KSA. Serum 25(OH)D data were available for subjects older than 1 and above years from 2017 to 2021. The database represents new cases visiting King Khalid Hospital for a biomarker screening program, with unique medical conditions, school projects for vitamin D, and community screening registry from a representative population of the Sudair district, KSA from 2017 to 2021. To ensure no duplicate cases were included and to maintain data integrity, each participant, regardless of entry point, was assigned a unique ID. This study acknowledges the presence of selection bias, as the participants may not represent the general population. Additionally, we were unable to determine the exact frequency of the screening programs conducted over the 5 years or identify the specific years in which they took place. These factors may impact the generalizability of the findings and have been considered as limitations in the analysis.

### 2.2 Procedure

To maintain the specimens’ integrity, blood samples were drawn into plain tubes, centrifuged, and processed all on the same day. A calibration curve was created for every analytical run to guarantee accuracy, and the analyzer was calibrated using approved reference materials. To preserve assay reliability, strict quality control procedures were followed. To monitor assay performance, internal quality control involved running control samples with known [25(OH)D] concentrations alongside each batch of test samples. The limit of detection and possible cross-reactivity with other vitamin D metabolites were used to evaluate the analytical sensitivity and specificity. To guarantee reproducibility, duplicate samples were examined in each run, and intra- and inter-assay variability was computed. The accuracy and consistency of the [25(OH)D] concentration measurements were guaranteed throughout the investigation by these extensive quality control procedures. Routine maintenance, including cleaning, reagent replacement, and system checks, was crucial to keep the analyzer in optimal condition. Proper storage and handling of reagents, strict adherence to expiry dates, and meticulous preparation according to guidelines were followed to prevent degradation. Routine performance checks of the system’s optics and fluidics were carried out, and any errors or abnormal results were promptly addressed to maintain consistent accuracy.

The concentration of [25(OH)D] was measured using ROCH COBAS e-411 analyzer where the principle of the test is a competitive protein binding immunoassay with electrochemiluminescence (ECLIA) detection. The Roche Cobas e-411 analyzer is a diagnostic instrument developed by Roche Diagnostics. It is designed for immunoassay testing, enabling the quantitative measurement of various biomarkers in patient samples. The Cobas e-411 analyzer is known for its efficiency, accuracy, and versatility in clinical laboratories (29). It utilizes the electrochemiluminescence (ECL) technology for precise and reliable results (30). The user-friendly interface and high-throughput capabilities of the Roche Cobas e-411 contribute to its popularity in clinical settings for routine diagnostic testing (31).

### 2.3 Vitamin D categories

The status of [25(OH)D] was categorized as ≥30 ng/mL (≥75 nmol/L) as sufficient, 20–29 ng/mL (≥50–<75 nmol/L) as insufficient, ≥7–<20 ng/mL (≥17.5–<50 nmol/L) as deficiency and <7 ng/mL (<17.5 nmol/L) considered as severe Vitamin D deficiency. The following formula was used to convert ng/ml into nmol/L (nmol/L = 2.469 X ng/ml) (32). Hypovitaminosis definition is complicated by [25(OH)D] assay variation. A systematic review of the global representation of [25(OH)D] concentrations as deficiency, insufficiency, sufficiency, and possible harm at <20 ng/mL, 21–29 ng/mL, 30–100 ng/mL, and >100 ng/mL, respectively (33). The Institute of Medicine established public health guidelines for healthy individuals, defining deficiency at <20 ng/mL, insufficient at 21–29 ng/mL, and sufficient at 30–100 ng/mL (34). However, most researchers, physicians, and government agencies found these two stand cut-off points confusing and needed identical interpretive cut-points. National agencies usually base their guidelines on foreign ones. The Netherlands, being more conservative, adopted the UK 1998 Committee on Medical Aspects of Food and Nutrition Policy recommendations to define a [25(OH)D] concentration of 10 ng/mL (25 nmoL/L) as the risk of rickets and osteomalacia for people aged 0–70 (35). This study utilized widely recognized cut points to categorize vitamin D concentrations, adopting <20 ng/mL to define deficiency and a more conservative threshold of <7 ng/mL to delineate severe deficiency. This severe form of deficiency is often defined variably, with thresholds ranging from less than 5 ng/mL to 10 ng/mL in different studies (36–38). In our study, we defined vitamin D deficiency at a threshold of 20 ng/mL and severe deficiency at 7 ng/mL. Additionally, we included a severe deficiency threshold of 10 ng/mL to facilitate the inclusion of our data in meta-analyses, allowing for broader comparisons and more comprehensive evaluations across various studies (Supplementary material). Based on the date of blood sampling for serum 25(OH)D, the season was plotted to determine the seasonal variation of serum 25(OH)D among the studied participants. Hot summers and mild winters are the two primary seasons of Saudi Arabia’s desert environment, with autumn and spring serving as transitional times. Summer lasts from June to August, with daytime highs sometimes approaching or surpassing 45°C. Winter typically lasts from December to February, when temperatures can dip as low as 15°C. The seasons of spring (March to May) and autumn fall (September to November) are transitional.

### 2.4 Statistical analysis

**Figure 1:**
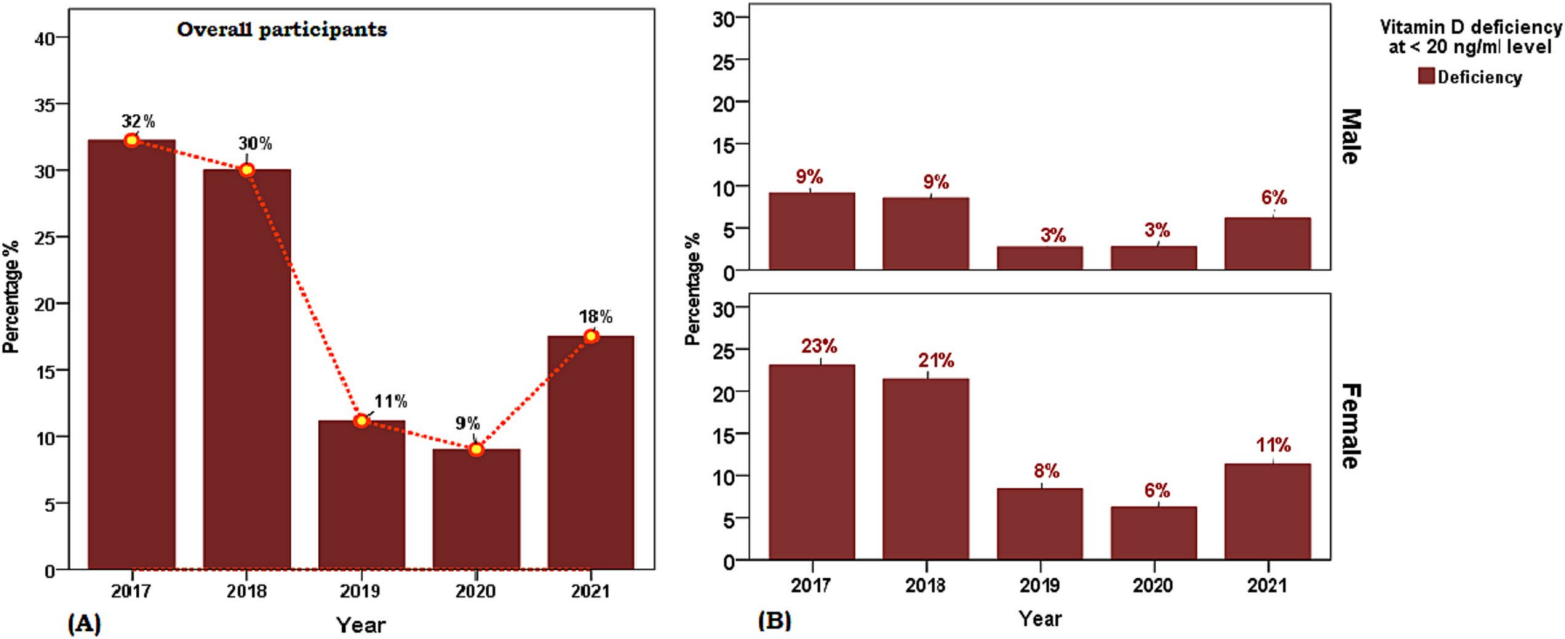
Weighted prevalence of serum [25(OH)D] concentration of <7 (severe deficiency), 7 to <20 (deficiency), 20 to 29 (insufficiency), and 30 and above in units ng/ml (sufficient) in Saudi Arabian population aged ≥ 1 years between 2017 and 2021. **(A)** Overall participants and **(B)** Sex-based distribution. The reference lines indicate 30 ng/mL concentration (green), 20 ng/mL concentration (yellow), and 7 ng/mL concentration (red).

Data were descriptively reported as mean, standard deviation, standard error, and frequency with percentages for continuous and categorical variables correspondingly. Independent t-test and ANOVA were employed to assess the mean difference. While Chi-square or Fisher Exact test were used with frequency distribution where appropriate. The association between time (2017–2021) and serum [25(OH)D] ng/ml was examined using regression analysis. A linear trend of mean serum [25(OH)D] ng/ml concentration as a dependent variable from 2017 to 2021 years was analyzed using a linear regression model, by treating the years as continuous independent predictors when other variables were adjusted. Analysis was separated by gender and age groups. The weighted prevalence of serum [25(OH)D] concentration categorized as >30 ng/mL (sufficient), 20–29 ng/mL (insufficient), <20 ng/mL (deficiency), and <7 ng/mL as (severe deficiency) of vitamin D was calculated by dividing the weighted number of participants with different concentration of vitamin D by the weighted total number of people in the study (Figure 1). We categorized age into decades and also as >18 years (children and adolescents), 19–65 years (adults), and >65 years (older) to explore year trends and distribution of serum [25(OH)D] ng/ml concentration. Statistical comparison employed difference of means or proportions with a 95% confidence interval. The threshold of significance was set at <0.05. Statistical analysis was performed using Statistical Package for Social Sciences (SPSS) version 21.0.

## 3 Results

### 3.1 Demographic characteristics

The characteristics of the 22,335 participants of the retrospective cohort cross-sectional study included in the final analysis are presented in Table 1. The sex distribution of the study population was 30.1% males and 69.9% females and the mean age between sex groups was statistically similar (t = 0.253, *p* > 0.05). The overall mean serum [25(OH)D] concentration was 25.6 ± 14.1 ng/mL with no sex disparity (males 25.7 ng/mL vs. females 26.6 ng/mL, t = 0.31, *p* > 0.05) and the frequency distribution of the proportion of age group decades was observed to represent a symmetrical bell-shaped spread out. A statistically significant difference was observed in the proportion of year-wise (2017 to 2021) weighted prevalence of vitamin D deficiency category (7 to <20 ng/mL) and between sexes for the year 2018 (male 33.1% vs. female 26.3%, χ2 = 22.46, *p* < 0.001, phi = 0.65), 2019 (male 18.8% vs. female 21.2%, χ2 = 16.18, *p* < 0.001, phi = 0.64), 2020 (male 18.6% vs. female 20.9%, χ2 = 16.43, *p* < 0.001, phi = 0.70) and weighted prevalence of vitamin D severe deficiency category (<7 ng/mL) between sexes 2018 (male 22.4% vs. female 37.2%), 2019 (male 4.4% vs. female 6.4%), 2021 (male 9.8% vs. female 6.7%) (Figure 1). The distribution of sex between the years (2017–2021) (df 4, χ2 = 187.1, *p* < 0.05, phi = 0.56) was statistically different between the years 2017 to 2021.

**Table 1:** Demographic characteristics.

Parameter	Overall *n* (%)	Sex *n* (%)				χ^2^/t
		Males	95% CI	Females	95% CI	
*N*	22,335 (100)	6,720 (30.1)	29.5, 30.7	15,615 (69.9)	69.3, 70.5	
^t^Age (years)	41.88 ± 16.9	41.92 ± 18.8	41.5, 42.4	41.86 ± 16.13	41.6, 42.1	0.253
^a^Age group (decades)						
1–9 years	652 (2.9)	337 (51.7)	47.8, 55.6	315 (48.3)	44.4, 52.2	380.8^*^
10–19 years	1394 (6.2)	514 (36.9)	34.4, 39.5	880 (63.1)	60.5, 65.6	
20–29 years	3427 (15.3)	913 (26.6)	25.3, 28.2	2514 (73.4)	71.8, 74.7	
30–39 years	4649 (20.8)	1362 (29.3)	28.0, 30.7	3287 (70.7)	69.3,72.0	
40–49 years	4639 (20.7)	1161 (25)	23.8, 26.4	3478 (75)	73.6, 76.2	
50–59 years	4163 (18.6)	1126 (27)	25.7, 28.4	3037 (73)	71.6, 74.3	
60–69 years	2235 (10)	861 (38.5)	36.4, 40.5	1374 (61.5)	59.5, 63.6	
70–79 years	841 (3.8)	315 (37.5)	34.0, 40.7	526 (62.5)	59.3, 66	
≥80 years	335 (1.5)	131 (39.1)	33.9, 44.1	204 (60.9)	55.9, 66.1	
^t^25(OH)D (ng/ml)	25.6 ± 14.1	25.6 ± 13.7	25.3, 25.9	25.58 ± 14.2	14.1, 14.4	0.31
^a^Vitamin D category						
Sufficient concentration	7310 (32.7)	2137 (29.2)	28.1, 30.3	5173 (70.8)	69.7, 71.9	
Insufficient concentration	6274 (28.1)	2010 (32)	30.8, 33.2	4264 (68)	66.8, 69.2	
Deficiency	8014 (35.9)	2397 (29.9)	28.9, 30.8	5617 (70.1)	69.2, 71.1	
Severe deficiency	737 (3.3)	176 (23.9)	20.8, 27.0	561 (76.1)	73.0, 79.2	27.5^*^
^a^Year						
2017	5408 (24.2)	1559 (28.8)	27.6, 30.0	3849 (71.2)	70.0, 72.4	187.1^*^
2018	5360 (24)	1542 (28.8)	27.5, 30.0	3818 (71.2)	70.0, 72.5	
2019	3912 (17.5)	1055 (27)	25.6, 28.6	2857 (73)	71.5, 74.4	
2020	3320 (14.9)	1112 (33.5)	31.7,35.2	2208 (66.5)	64.8, 68.3	
2021	4335 (19.4)	1452 (33.5)	32.1, 34.8	2883 (66.6)	65.2, 67.9	

Values are presented as proportion, percentage (%) or mean, ± standard deviation, differences indicated by ^a^Chi-square test was used for categorical variables, ^t^student t-test for continuous variables. CI, confidence interval; 25(OH)D; 25-hydroxyvitamin D, Vitamin D category; sufficient concentration (≥30 ng/mL), Insufficient concentration (20–29 ng/mL), deficiency (<20 to 7 ng/mL), and severe deficiency (<7 ng/mL). ^*^Indicates significant differences and *p* < 0.05 was considered significant.

### 3.2 Prevalence of vitamin D deficiency, and trends

**Figure 2:**
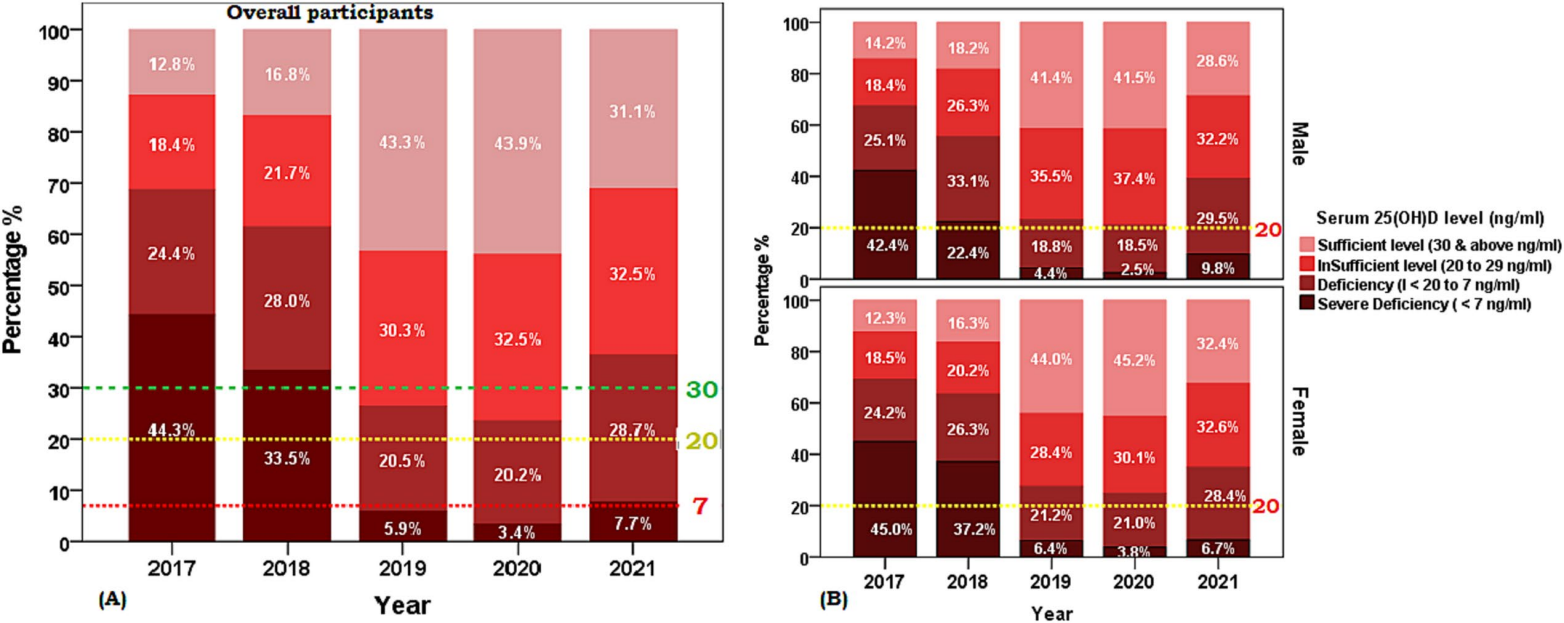
Weighted prevalence of serum [25(OH)D] concentration of <7, 7 to <20, 20 to 29, and 30 and above (ng/ml) by age groups of the Saudi Arabian population. **(A)** Overall participants and **(B)** Sex-based distribution. The reference lines indicate 30 ng/mL concentration (green), 20 ng/mL concentration (yellow), and 7 ng/mL concentration (red).

Table 2 presents the mean [25(OH)D] concentrations across age groups and years, stratified by sex. Notable findings include significant variations in [25(OH)D] concentration across age groups, with the highest mean concentration observed in the groups of 1–9 and 70–79 years (30.51 ± 15.6 ng/mL and 30.6 ± 15.2, respectively) and the lowest in the 10–19 years’ group (21.1 ± 11.9 ng/mL). Significant differences were observed across all age groups (*p* < 0.001) and between sex difference was observed in mean [25(OH)D] concentrations of age groups 10–19 years and 60–69 years. Figure 2 illustrates the proportion of vitamin D status of participants stratified by age group with sex subgroups. Additionally, the analysis by year reveals significant fluctuations, with the highest mean concentration in 2020 (31.36 ± 15.0 ng/mL) and the lowest in 2017 (20.87 ± 12.1 ng/mL) (Table 2). The overall trend of annual mean serum 25 (OH)D concentration measured from 2017 to 2020 showed an increasing trend (20.87 to 31.36) but a decline was observed between 2020 and 2021 (31.36 to 26.32 ng/mL) and the same trend was observed in both sexes. The linear regression analysis found a statistically significant (*p* < 0.001) positive slope for overall vitamin D concentration, as well as the concentrations for male, and female groups (1.1 ± 0.6, 0.1 ± 0.8, and 0.8 ± 0.9 respectively), suggesting an increasing trend in [25(OH)D] concentrations over the study period. *Post-hoc* analysis reveals specific age and year subgroups with significant differences.

**Table 2:** Mean 25(OH)D concentration (ng/ml) across age group and years (2017 to 2021) according to sex.

Variable	Overall mean (±)	Sex		*p* values
		Males (*n* = 6,720)	Female (*n* = 15,615)	
Age group				
1–9 years^A^	30.51 ± 15.6^BCDEF^	30.45 ± 15.7^BCDEF^	30.57 ± 15.6^BCDEF^	0.9
10–19 years^B^	21.13 ± 11.9^ADEFGHI^	22.20 ± 11.8^AEFGHI^	20.51 ± 12.1^ADEFGHI^	**0.01**
20–29 years^C^	22.20 ± 13.5^ADEFGHI^	22.23 ± 13.2^AEFGHI^	22.2 ± 13.6^ADEFGHI^	0.9
30–39 years^D^	24.18 ± 13.3^ABCEFGHI^	23.86 ± 12.9^AEFGH^	24.31 ± 13.4^ABCEFGHI^	0.3
40–49 years^E^	25.82 ± 13.7^ABCDFGHI^	25.77 ± 13.4^ABCDGH^	25.84 ± 13.9^ABCDFGHI^	0.8
50–59 years^F^	27.50 ± 14.2^ABCDEGH^	27.32 ± 13.6^ABCDH^	27.57 ± 14.5^ABCDEGH^	0.6
60–69 years^G^	28.75 ± 14.5^BCDEFH^	27.93 ± 13.7^BCDE^	29.27 ± 14.9^BCDEF^	**0.03**
70–79 years^H^	30.60 ± 15.2^BCDEFG^	30.22 ± 14.9^BCDEF^	30.83 ± 15.3^BCDEF^	0.6
≥80 years^I^	28.91 ± 16.0^BCDE^	27.63 ± 14.8^BC^	29.73 ± 16.7^BCDE^	0.2
***p* value**	**<0.001**	**<0.001**	**<0.001**	
**Slope**	**1.14** ± 0.6, <0.001	**0.11** ± 0.8, <0.01	**0.77** ± 0.9, <0.001	
Year				
2017^A^	20.87 ± 12.1^BCDE^	21.25 ± 12.4^CDE^	20.72 ± 12.0^BCDE^	0.2
2018^B^	22.37 ± 12.8^ACDE^	22.43 ± 12.1^CDE^	22.35 ± 13.1^ACDE^	0.8
2019^C^	30.89 ± 14.7^ABE^	30.51 ± 13.7^ABE^	31.03 ± 15.1^ABE^	0.3
2020^D^	31.36 ± 15.0^ABE^	31.21 ± 14.8^ABE^	31.43 ± 15.1^ABE^	0.7
2021^E^	26.32 ± 13.2^ABCD^	25.99 ± 13.3^ABCD^	26.48 ± 13.2^ABCD^	0.3
***p* value**	**<0.001**	**<0.001**	**<0.001**	

The superscript A, B, C, D, E, F, G, H, I indicates significant difference by multiple comparisons between the subgroups of variable column using *post-hoc* analysis with alpha (α) at <0.05 level. Slope and *p*-value are calculated using linear regression analysis. Bold values indicate significant difference.

### 3.3 Age group and sex-specific stratification of vitamin D status

**Figure 3:**
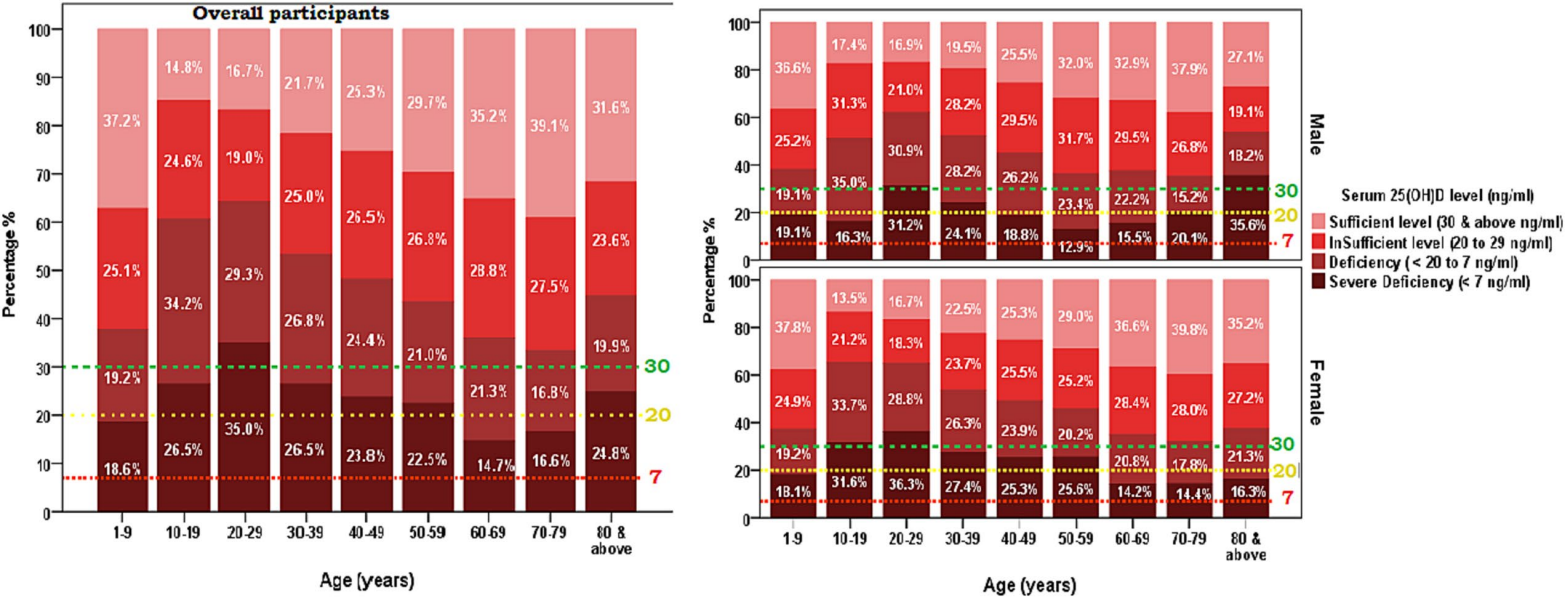
The changes in the prevalence of vitamin D deficiency from 2017 to 2021, vitamin D deficiency was defined as the serum 25-hydroxyvitamin D concentration of less than 20 ng/mL concentration **(A)** overall participants **(B)** Sex-based distribution.

The age and gender-adjusted mean [25(OH)D] concentrations for each age group (<18 years, 19–65 years, and >65 years) from 2017 to 2021 are shown in Table 3. Between 2017 and 2020, the corrected mean concentrations for each year show an increasing trend, rising from 20.8 to 31.4 ng/mL but decreased to 26.54 ng/mL in 2021, a similar pattern was observed in the distribution of the proportion of participants with deficiency at <20 ng/mL (Figure 3). Regression analysis also showed a significant difference across groups for the trend (increasing) with the highest increment observed in participants aged less than 18 years with 1.2 ng/mL and the smallest increment of 0.88 ng/mL was observed between 19 and 65 years. Sex-specific stratification reveals that during the investigation, both male and female groups had comparable rising tendencies, with significant positive slopes (1.1 and 1.0, respectively; *p* < 0.001).

**Table 3:** Age and gender adjusted 25(OH)D concentration (ng/ml) from 2017 to 2018 according to age category.

Variable	Overall (SEM)	Sex^		Age category^Ϯ^		
		Males (*n* = 6,720)	Female (*n* = 15,615)	<18 years	19–65 years	>65 years
Year						
2017	20.8 (0.18)	21.2 (0.33)	20.6 (0.21)	21.4 (0.66)	20.7 (0.19)	21.5 (0.64)
2018	22.3 (0.17)	22.4 (0.31)	22.2 (0.22)	23.1 (0.67)	21.7 (0.2)	27.7 (0.65)
2019	30.8 (0.21)	30.49 (0.40)	30.9 (0.25)	30.3 (0.78)	30.2 (0.23)	37.54 (0.74)
2020	31.4 (0.23)	31.2 (0.39)	31.6 (0.28)	27.8 (0.86)	31.2 (0.25)	38.2 (0.96)
2021	26.54 (0.20)	26.2 (0.34)	26.7 (0.24)	22.3 (0.65)	26.47 (0.22)	30.7 (0.83)
Slope	1.1 (0.3)	1.0 (0.2)	1.01 (0.1)	1.2 (0.3)	0.9 (0.1)	1.0 (0.2)
*p* value	<0.001	<0.001	<0.001	<0.001	<0.01	<0.001

Data are presented as mean and SEM-Standard error of mean. Slope and *p* value are calculated using linear regression analysis. ^Indicates adjustment for age and ^Ϯ^indicates adjustment for gender.

#### 3.3.1 Structure of variability

The age distribution in relation to the Vitamin D level [25(OH)D] showed a quasi-U-shaped relationship with the lowest point in the curve from 10 to 29 years. A significant correlation was found between age and the mean [25(OH)D] ng/ml [Vitamin D] for the overall participants with r = 0.14, *p* < 0.05. Supplementary Figure 1 shows that the long-range correlations followed a quasi-U-shaped distribution pattern between ages 10 to 29 years. There is a significant decrease in the strength of [25(OH)D] ng/ml correlations between 10 and 29 years of age, and a gradual increase in the strength between 29 and 79 years of age (Supplementary material).

Table 4 presents an overview of the proportion of vitamin D deficiency (<20 to 7 ng/mL) and severe deficiency (<7 mg/mL) among all the tested men from 2017 to 2021, classified by sex and age categories. The prevalence of vitamin D deficiency in men was highest in the year 2017 (44.5%) and 2018 (44.7%), with a substantial decrease in the proportion of prevalence in the following years, 2019 (22.2%) and 2020 (21.6). Again, the prevalence proportion of vitamin D deficiency (7 to <20 ng/mL) among men rose higher to 36.1% in the year 2021. A similar trend of prevalence proportion of vitamin D deficiency was observed among females with the higher prevalence in 2017 (44.8%) and 2018 (43.5). Then, the trend decreased to 25.2 and 24.4, in the year 2019 and 2020, respectively. In the year 2021, the prevalence of vitamin D deficiency (7 to <20 ng/mL) rose to 33.7% among females.

**Table 4:** Sex-wise and age category-wise distribution of vitamin D deficiency [25(OH)D] < 20 ng/mL and severe deficiency [25(OH)D] < 7 ng/mL from year 2017 to 2021.

Year (total *N* tested)	Sex	Total sample	Vitamin D deficiency [25(OH)D] < 20 ng/mL, *n* (%)	Vitamin D severe deficiency [25(OH)D] < 7 ng/mL, *n* (%)
2017 (*n* = 5408)	Male	1,559	694 (44.5)	108 (6.9)
	Female	3,879	1,725 (44.8)	295 (7.7)
2018 (*n* = 5360)	Male	1,542	705 (45.7)	44 (2.9)
	Female	3,818	1,662 (43.5)	216 (5.7)
2019 (*n* = 3912)	Male	1,055	234 (22.2)	5 (0.5)
	Female	2,857	719 (25.2)	20 (0.7)
2020 (*n* = 3320)	Male	1,112	240 (21.6)	3 (0.3)
	Female	2,208	538 (24.4)	9 (0.4)
2021 (*n* = 4335)	Male	1,452	524 (36.1)	16 (1.1)
	Female	2,883	973 (33.7)	21 (0.7)
*p* value			<0.001	<0.001

Year (total *N* tested)	Age category	*N*	<20 ng/mL *n* (%)	<7 ng/mL *n* (%)
2017 (*n* = 5,408)	<18 years	413	186 (45)	24 (5.8)
	19–65 years	4531	2,014 (44.4)	360 (7.9)
	>65 years	464	219 (47.2)	19 (4.1)
2018 (*n* = 5,360)	<18 years	415	183 (44.1)	15 (3.6)
	19–65 years	4485	2,046 (45.6)	234 (5.2)
	>65 years	460	138 (30)	11 (2.4)
2019 (*n* = 3,912)	<18 years	301	76 (25.2)	03 (1)
	19–65 years	3257	845 (25.9)	21 (0.6)
	>65 years	354	32 (9)	1 (0.3)
2020 (*n* = 3,320)	<18 years	250	88 (35.2)	01 (0.4)
	19–65 years	2861	672 (23.5)	10 (0.3)
	>65 years	209	18 (8.6)	1 (0.5)
2021 (*n* = 4,335)	<18 years	434	218 (50.2)	05 (1.2)
	19–65 years	3622	1,232 (34)	27 (0.7)
	>65 years	279	47 (16.8)	05 (1.8)
*p* value			<0.001	<0.001

Data presented are frequency and percent. *Indicate *p* value for trend (Chi-square-MH test linear to linear association).

On the other hand, the proportion of severe deficiency was observed with a decreasing trend from the year 2017 to 2020 in both men and women (2017 [6.9 and 7.7], 2018 [2.9 and 5.7], 2019 [0.5 and 0.7], 2020 [0.3 and 0.4]). However, the prevalence marginally increased in both men and women in the year 2021, at 1.1 and 0.7, respectively. The age-group-specific distribution of vitamin D deficiency (<20 to 7 ng/mL) and severe deficiency (<7 mg/mL) showed that among 434 tested subjects in the age group < 18 years, about ½ (50.2%) of them were observed with 25(OH)D ng/ml level between 7 and <20, which is the highest proportion of vitamin deficiency for <18 years age-group across years 2017 to 2021. The proportion of severe vitamin D deficiency was observed to decrease from the year 2017 to 2020 in each age group. The *p*-values for the trend analysis do not reach statistical significance in cases of severe insufficiency (Figure 3).

**Figure 4:**
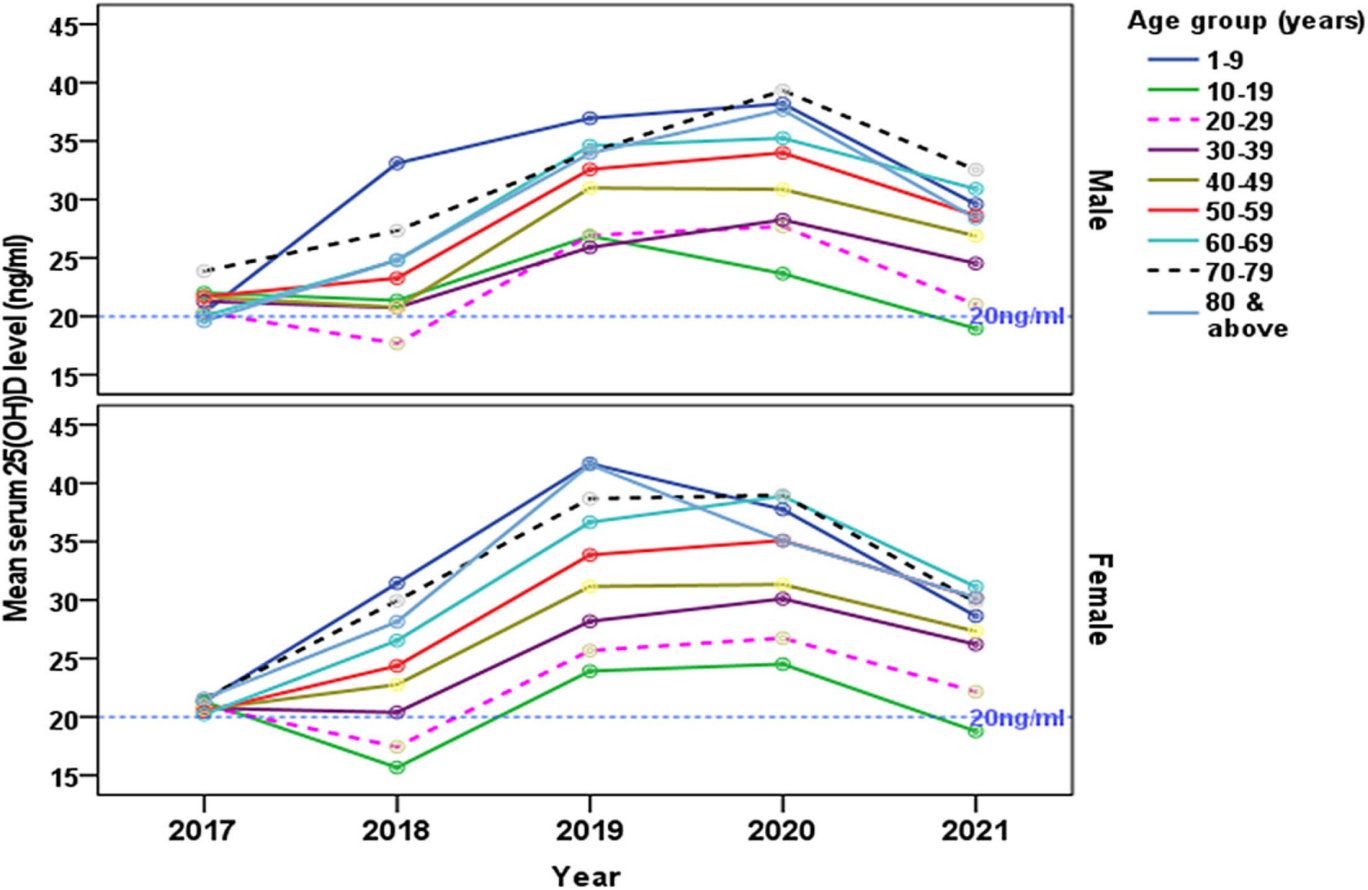
The changes of gender-specific mean serum 25-hydroxyvitamin D [25(OH)D] concentration (ng/ml) by age groups from 2017 to 2021. The reference line indicates (20 ng/mL) level.

The temporal changes of gender-specific mean serum concentration of [25(OH)D] from 2017 to 2021 are presented by age groups (Figure 4). The absolute decrease in serum [25(OH)D] concentration between 2017 and 2018 was highest in those aged 10–19 years and 20–29 years among females, and those aged 20–29 years in males. A gradual rise in the trends of serum [25(OH)D] was observed from 2018 to 2020 in all age groups in both male and female. There is a notable decrease in the trend of concentration of the serum [25(OH)D] from 2020 onwards.

#### 3.3.2 Seasonal variation of serum 25(OH)D concentration, Saudi Arabia

A one-way ANOVA was conducted to determine whether mean serum 25(OH)D concentrations (ng/ml) differed across four seasons (Autumn, Spring, Summer, Winter). The means ± SD (95% CI) were: Autumn, 25.90 ± 13.96 (25.46–26.35); Spring, 25.68 ± 14.09 (25.23–26.12); Summer, 25.49 ± 14.08 (25.18–25.79); Winter, 25.53 ± 14.16 (25.18–25.87); and Overall, 25.60 ± 14.08 (25.42–25.79). The ANOVA yielded an *F*-value of 0.853 (*p* = 0.465), indicating no statistically significant differences among seasons, and Tukey *post hoc* comparisons similarly revealed no significant pairwise differences (*p* > 0.05) (Tables 5, 6).

**Table 5:** Frequency distribution of participants across years (2017–2021) and seasons.

Year	Seasons, number ± percentage			
	Autumn	Spring	Summer	Winter
2017	888 (16.4)	945 (17.5)	2,076 (38.4)	1,499 (27.7)
2018	916 (17.1)	921 (17.2)	1,957 (36.5)	1,566 (29.2)
2019	683 (17.5)	693 (17.7)	1,438 (36.8)	1,098 (28.1)
2020	563 (17)	553 (16.7)	1,236 (37.2)	968 (29.2)
2021	733 (16.9)	757 (17.5)	1,595 (36.8)	1,250 (28.8)

**Table 6:** Year-wise (2017–2021) change in the mean concentration of serum 25(OH)D in each season.

Year	Seasons, mean ± SD (ng/mL)				*p* value
	Autumn	Spring	Summer	Winter	
2017	21.41 ± 11.75	20.89 ± 12.26	21.07 ± 12.38	20.28 ± 11.81	0.116
2018	22.63 ± 12.48	22.58 ± 12.97	22.35 ± 12.92	22.12 ± 12.93	0.740
2019	30.70 ± 14.70	31.15 ± 14.71	30.55 ± 14.66	31.29 ± 14.86	0.587
2020	32.36 ± 15.30	30.90 ± 15.13	31.06 ± 14.86	31.42 ± 14.93	0.318
2021	25.99 ± 13.12	26.59 ± 12.97	26.22 ± 13.31	26.46 ± 13.25	0.803

## 4 Discussion

The key findings of this retrospective cross-sectional study, conducted over 5 years (2017–2021) and including a large sample of 22,335 participants, revealed that 67.3% of participants in the central area of KSA had <30 ng/mL of 25(OH)D. Female participants, particularly those aged 10–19 and 20–29 years, had a higher prevalence of vitamin D insufficiency. Over the years, there was a notable decrease in the prevalence of insufficiency deficiency (<20 ng/mL), dropping from 32% in 2017 to 9% in 2020. However, there was a little slight increase to 18% in 2021.

### 4.1 Period prevalence of 25-hydroxyvitamin D deficiency (2017–2021)

The period prevalence of vitamin D deficiency in the central region of KSA (SA) was 67.1% with highest prevalence of severe vitamin D deficiency in 2017 (see Figure 1). In line with our results a systematic review of 16 relevant studies conducted between 2008 and 2015 in the Saudi population, involving 20,787 patients, demonstrated a substantial prevalence of Vitamin D deficiency at 63.5% (95% CI: 53.3, 73.7), with females constituting 62% of the affected individuals (16). Similarly, a study conducted from 2013 to 2014 among people living in the Eastern region of SA, revealed a significant prevalence of vitamin D deficiency, and the highest prevalence of 49.5% in students and 44% in employees (18). A recent study spanning from 2008 to 2017 in the central region of KSA, involving 7,360 participants, reported a significant reduction in vitamin D deficiency, showcasing an overall prevalence of 73.2% (21).

Our study revealed a consistent decreasing trend in vitamin D deficiency, dropping from 32% in 2017 to 9% in 2020, followed by an increase of 18% in 2021. The declining trend observed from 2017 to 2020 can be attributed to the success of public health campaigns addressing vitamin D deficiency in KSA. As per the directives from the Ministry of Health in 2013, companies involved in the production of milk and milk products, such as Almarai, are adhering to the practice of fortifying their products with vitamin D. Specifically, fortified milk with approximately 400 IU/L of vitamin D, along with other milk-derived products are now commonly available (39). Conversely, the notable increase in 2021 may be associated to the COVID-19 pandemic, which necessitated individuals to stay indoors, resulting in reduced exposure to sunlight and limited physical activity (40).

### 4.2 Age-specific trends in 25-hydroxyvitamin D deficiency

This study showed the highest prevalence of severe [25(OH)D] deficiency was observed among the age group of 20 to 29 years old with the mean concentration below 20 ng/mL (deficiency) found in females in 2017–2018 (Figures 2, 3). Similar to our findings, a 2009 study conducted in the Eastern region of KSA, involving 139 healthy Saudi adults, reported an average [25(OH)D] concentration of approximately 10.1 ng/mL in men and 9.9 ng/mL in women (20).

Our results indicate that the age groups, particularly females aged 10–19 and 20-29-year-old (Figure 4), exhibited a mean concentration of [25(OH)D] deficiency below 20 ng/mL, which subsequently improved, with none falling below the 20 ng/mL cutoff score. However, it is noteworthy that both male and female age groups (10–19 years old) displayed concentrations below 20 ng/mL in 2021. Comparable to our findings, a study conducted in the central region of the KSA from 2008 to 2017 reported improvement in participants aged 18–40 years, with deficiency decreasing from 87.1% to 64.7%, and in those aged >40 years, where deficiency decreased from 86.2% to 45.7% (21).

These positive outcomes can be attributed to the successful health policies implemented by the government of KSA from 2010 through 2020. These policies included the supplementary addition of cholecalciferol (vitamin D3) to daily consumed food items such as fresh milk, yogurt, powdered milk, cheese, ready-to-eat breakfast cereals, and orange juice (39). It is important to note that the decrease in the mean concentration of 25-Hydroxycholecalciferol [25(OH)D] is often multifactorial, involving factors such as reduced kidney efficiency with advancing age, physical activity, dietary patterns, genetic factors, skin pigmentation, underlying medical conditions, and genetics (41). Consequently, further studies are warranted to thoroughly explore the impact of these factors on the mean concentration of vitamin D deficiency.

### 4.3 Disparity in [25(OH)D] deficiency across genders

Among adolescents, females exhibited a higher prevalence of vitamin D deficiency compared to males with the highest prevalence in 2017 (23%) and 2018 (21%) (Figure 4). Similarly, gender-specific trends showcased a decreasing trend in [25(OH)D] deficiency in females from 80.1% to 69.6% and in males from 93.2% to 49.3% (21). Similarly, a study conducted in the Eastern region involving 139 adult participants reported a consistently lower serum [25(OH)D] concentration (10.1 ng/mL for males and 9.9 ng/mL for females) despite adequate exposure to sunlight and recommended dairy product intake (20). Despite adequate sunlight exposure, over 65% of participants and more than 90% reported sufficient dairy product intake, serum [25(OH)D] concentrations were consistently low (10.1 ng/mL for males and 9.9 ng/mL for females) (20). In KSA, despite the abundance of sunlight throughout the year, direct exposure to sunlight would be limited in the population, particularly among females, due to conservative clothing, cultural conventions limiting outdoor activities, and the hindrance to daytime activities caused by high daytime temperatures. Further limited balanced dietary practices that involve consuming fewer foods high in vitamin D and possible ignorance of the vitamin’s significance exacerbate the problem. Thus these could explain the similarity in the vitamin D deficiency reported (39).

### 4.4 Temporal and seasonal variation of vitamin D deficiency (2017–2021)

Our results revealed a notable decrease in the prevalence of vitamin D deficiency (below 20 ng/mL) from 32% in 2017 to 9% in 2020, with a significant decrease in prevalence from 30% in 2018 to 11% in 2019 (see Figure 4). The observed positive trend in vitamin D concentration levels can be attributed to several factors, including increased awareness, public health campaigns, and potential changes in lifestyle or dietary habits that have encouraged greater exposure to sunlight and the consumption of vitamin D-fortified foods. However, the implementation of COVID-19 restrictions in 2021 seemingly contributed to a slight deviation in this declining pattern (40). The COVID-19 pandemic from 2019 to 2021 is reported to impact the dynamics of hypovitaminosis D due to lifestyle and environmental variables (42), but it is mostly associated with decreased sun exposure, which is the main source of ultraviolet-B (UVB)-induced vitamin D synthesis in the skin (43). Season dependent fluctuations in serum 25(OH)D concentrations among people has been reported in studies with deficiencies being observed especially during winter (44). Studies observed that this pattern is particularly more in the high latitude countries (>50°North or South) (45) with no sunlight of appropriate wavelength for synthesis of pre-vitamin D. At the same time, other factors like clothing style and sun avoidance behavior were also reported as determinants of vitamin D deficiencies in mid and low latitude countries.

Interestingly, our findings showed no significant seasonal differences in serum 25(OH)D levels, which contrasts with many studies in colder, higher-latitude countries where winter often brings a marked drop in vitamin D status. In Saudi Arabia, year-round sunshine and mild winter temperatures (around 18°C to 25°C) and the study area is located at the latitude of 25.72° N, may be sufficient for vitamin D skin synthesis, helping to offset such seasonal dips. Diet is another factor, as vitamin D can also come from fortified dairy products and fatty fish, though actual consumption patterns in Saudi Arabia could influence overall vitamin D status. Moreover, despite a high UV index throughout the year, lifestyle factors such as covering clothing, limited outdoor activity during extreme heat, and widespread use of sunscreen can reduce opportunities for natural vitamin D production. These findings highlight the complex interplay between environment, diet, and cultural practices, indicating a need for interventions—like education on safe sun exposure and dietary guidance—to address persistently low vitamin D levels in this population.

### 4.5 Limitations

While the study yields valuable insights, it is crucial to recognize certain limitations. These encompass the retrospective nature of the data analysis and the potential presence of unaccounted confounding factors. Additionally, the observational design of the study precludes the establishment of causation, underscoring the imperative for further research to unravel the intricate factors influencing vitamin D deficiency in the Saudi Arabian population. It is noteworthy that the study exclusively focuses on a central region of KSA, and caution must be exercised in generalizing the findings to the remaining 12 provinces in the country. Despite its limitations, an important strength of this study is the large sample size spanning 5 years, which provides a comprehensive understanding of the trends in vitamin D deficiency within the representative population. The dataset does not provide details on how individuals were selected for vitamin D investigations, nor does it specify the pathways through which they came for testing. Additionally, seasonal variation in vitamin D levels could not be assessed due to the absence of relevant data. Additionally, we were unable to determine the exact frequency of the screening programs conducted over the 5 years or identify the specific years in which they took place. This limitation could have led to inflated prevalence in some years and reduced prevalence in others. Moreover, only a single blood sample was collected from each participant, preventing any longitudinal assessment of vitamin D levels across different seasons, we could not reliably evaluate seasonal variations.

## 5 Conclusion

In conclusion, this cross-sectional study identified an overall period prevalence of 67.3% for 25(OH)D deficiency in the central region of KSA from 2017 to 2021. Vitamin D deficiency (<20 ng/mL) declined from 32% in 2017 to 9% in 2020, then rose to 18% in 2021. Females, especially those aged 10–19 and 20–29 years, were found to be the most affected group. These findings underscore the need for targeted, age-specific public health interventions to improve vitamin D status in high-risk groups. They also highlight the importance of continued efforts to address vitamin D deficiency at the population level. Future research endeavors should prioritize prospective studies to explore causative factors and assess the effectiveness of interventions in mitigating vitamin D deficiency in KSA. These findings hold implications for public health policy, emphasizing the ongoing necessity to address nutritional health in the population.

### 5.1 Recommendations and future studies

1. Develop and implement public health strategies tailored to specific age groups, with a particular focus on females aged 10–19 and 20–29 years old, to effectively address and improve vitamin D status.
2. Establish a systematic and continuous monitoring system for vitamin D deficiency trends on the national level, enabling the prompt identification of changes in prevalence and facilitating the adjustment of interventions as needed.
3. Prioritize and conduct prospective studies to comprehensively investigate the causative factors contributing to vitamin D deficiency in the Saudi population, providing valuable insights for targeted intervention strategies.
4. Rigorously evaluate the effectiveness of interventions designed to mitigate vitamin D deficiency, ensuring that evidence-based strategies are implemented for optimal outcomes.
5. Implement comprehensive educational programs to raise awareness about the importance of maintaining optimal vitamin D concentrations, especially among high-risk groups, fostering a culture of proactive health management.
6. Promote collaborative efforts among healthcare providers, researchers, and policymakers, fostering a multidisciplinary approach to comprehensively address and prevent vitamin D deficiency in KSA.

## Data Availability

The original contributions presented in the study are included in the article/Supplementary material, further inquiries can be directed to the corresponding author.
