# Supplementary material for: Prevalence and trends of vitamin D deficiency in a Saudi Arabian population: a five-years retrospective study from 2017 to 2021
Source: Front Public Health. 2025 Apr 28;13:1535980. doi: 10.3389/fpubh.2025.1535980 (PMC12066668; doi:10.3389/fpubh.2025.1535980)

**Additional tables**

**Table 1**: Distribution of vitamin D status in Saudi Arabia population from 2017 to 2021 (n = 22335)

| **Parameter** | **Overall**  **n (%)** | **Vitamin D category** | | | | χ^2^/t |
| --- | --- | --- | --- | --- | --- | --- |
|  |  | S (≥ 30 ng/ml) | IS (20-29 ng/ml) | D (<20 – 10 ng/ml) | SD (< 10 ng/ml) |  |
| N | 22335 (100) | 7310 (32.7) | 6274 (28.1) | 6721 (30.1) | 2030 (9.1) |  |
| Sex |  |  |  |  |  |  |
| Male | 6720 (30.1) | 2137 (31.8) | 2010 (29.9) | 2054 (30.6) | 519 (7.7) | 34.3* |
| Female | 15615 (69.9) | 5173 (33.1) | 4264 (27.3) | 4667 (29.9) | 1511 (9.7) |  |
| Age group (decades) |  |  |  |  |  |  |
| 1 – 9 years | 652 (2.9) | 297 (45.6) | 172 (26.4) | 147 (22.5) | 36 (5.5) | 819.7** |
| 10 – 19 years | 1394 (6.2) | 271 (19.4) | 387 (27.8) | 570 (40.9) | 166 (11.9) |  |
| 20 – 29 years | 3427 (15.3) | 835 (24.4) | 813 (23.7) | 1291 (37.7) | 488 (14.2) |  |
| 30 – 39 years | 4649 (20.8) | 1338 (28.8) | 1327 (28.5) | 1511 (32.5) | 473 (10.2) |  |
| 40 – 49 years | 4639 (20.7) | 1521 (32.8) | 1365 (29.4) | 1384 (29.8) | 369 (8) |  |
| 50 – 59 years | 4163 (18.6) | 1585 (38.1) | 1229 (29.5) | 1056 (25.4) | 293 (7) |  |
| 60 – 69 years | 2235 (10) | 927 (41.5) | 652 (29.2) | 521 (23.3) | 135 (6) |  |
| 70 – 79 years | 841 (3.8) | 397 (47.2) | 240 (28.5) | 160 (19) | 44 (5.2) |  |
| ≥ 80 years | 335 (1.5) | 139 (41.5) | 89 (26.6) | 81 (24.2) | 26 (7.8) |  |
| Year |  |  |  |  |  |  |
| 2017 | 5408 (24.2) | 1158 (21.4) | 1428 (26.4) | 1906 (35.2) | 916 (16.9) | 1975.8** |
| 2018 | 5360 (24) | 1295 (24.2) | 1438 (26.8) | 1896 (35.4) | 731 (13.6) |  |
| 2019 | 3912 (17.5) | 1833 (46.9) | 1101 (28.1) | 878 (22.4) | 100 (2.6) |  |
| 2020 | 3320 (14.9) | 1547 (46.6) | 983 (29.6) | 736 (22.2) | 54 (1.6) |  |
| 2021 | 4335 (19.4) | 1477 (34.1) | 1324 (30.5) | 1305 (30.1) | 229 (5.3) |  |

S – Sufficient level, IS – Insufficient level, D –Deficiency, SD – Severe Deficiency. ***** indicates significant differences and p < 0.05 was considered significant. ** indicates significant differences and p < 0.01 was considered significant.

**Table 2** Vitamin D Deficiency (< 20 ng/ml cut point) and severe deficiency (< 10 ng/ml cut point) from the year 2017 to 2021 according to Sex and Age

| **Year** | **Sex** |  | **Age category, n (%)** | | |
| --- | --- | --- | --- | --- | --- |
|  | **Males, n (%)**  **(n = 2573)** | **Females, n (%)**  **(n =6178)** | **< 18 years**  **(n = 168)** | **18 - 65 years**  **(n = 1758)** | **>65 years**  **(n = 104)** |
| **Vitamin D Severe Deficiency (25(OH)D) < 10 ng/ml** | | | | | |
| 2017 | 259 (28.3) | 657 (71.7) | 54 (5.9) | 796 (86.9) | 66 (7.2) |
| 2018 | 163 (22.3) | 568 (77.7) | 55 (7.5) | 649 (88.8) | 27 (3.7) |
| 2019 | 22 (22) | 78 (78) | 09 (9) | 90 (90) | 01 (1) |
| 2020 | 12 (22.2) | 42 (77.8) | 07 (13) | 45 (83.3) | 02 (3.7) |
| 2021 | 63 (27.5) | 166 (72.5) | 43 (18.8) | 178 (77.7) | 08 (3.5) |
| p value | 0.054 | | **< 0.001** | | |

Data presented are frequency and percent. *Indicate p value for trend (Chi-square-MH test linear to linear association).

**Additional figures**

**Figure 1** Long range correlation between mean 25(OH)D) concentrations and age group of participants from 10 to 29 years (n= 4821).


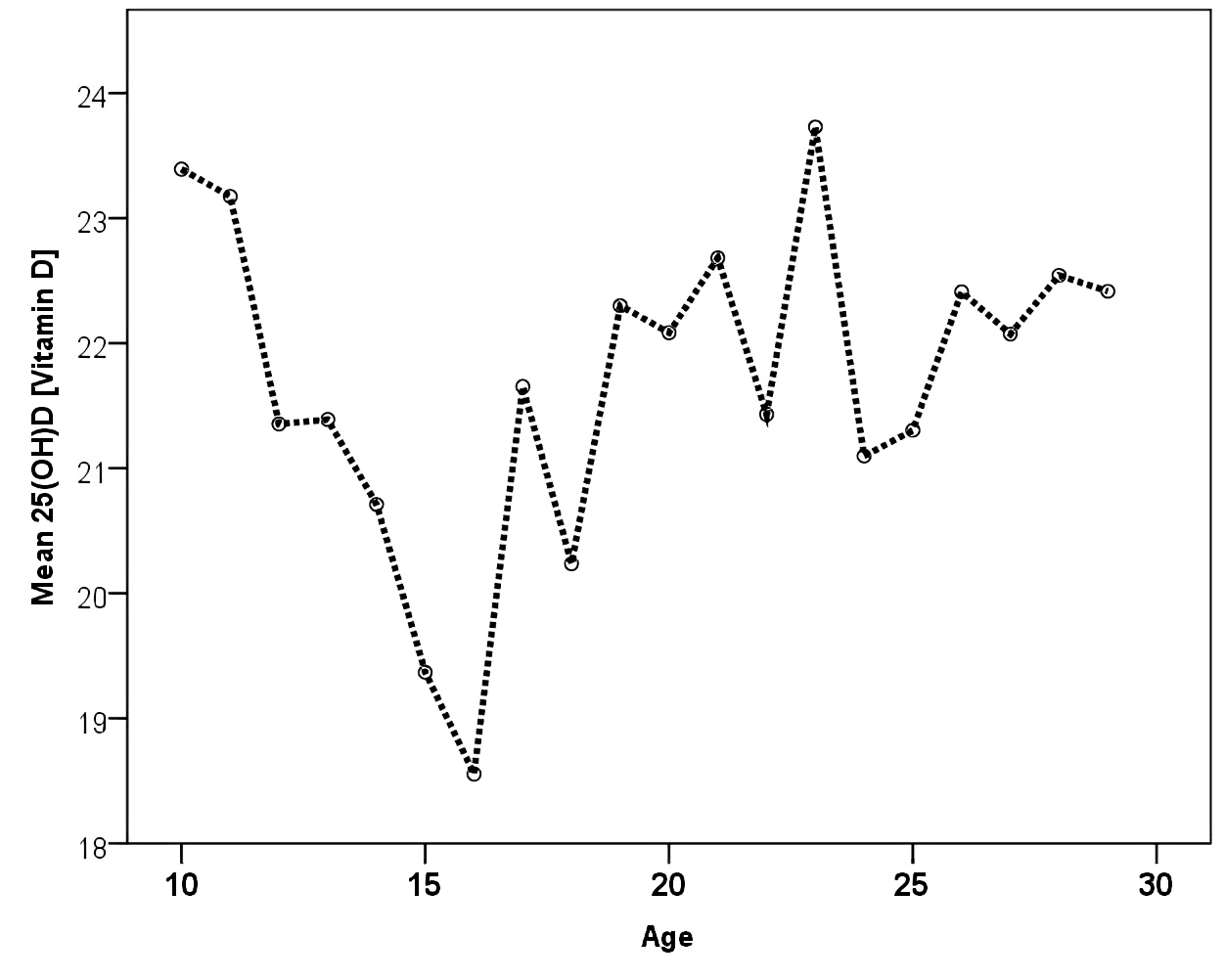


**Figure 2** Correlation between the age category and the mean value of 25(OH)D) ng/ml [Vitamin D] concentrations (n = 22330)


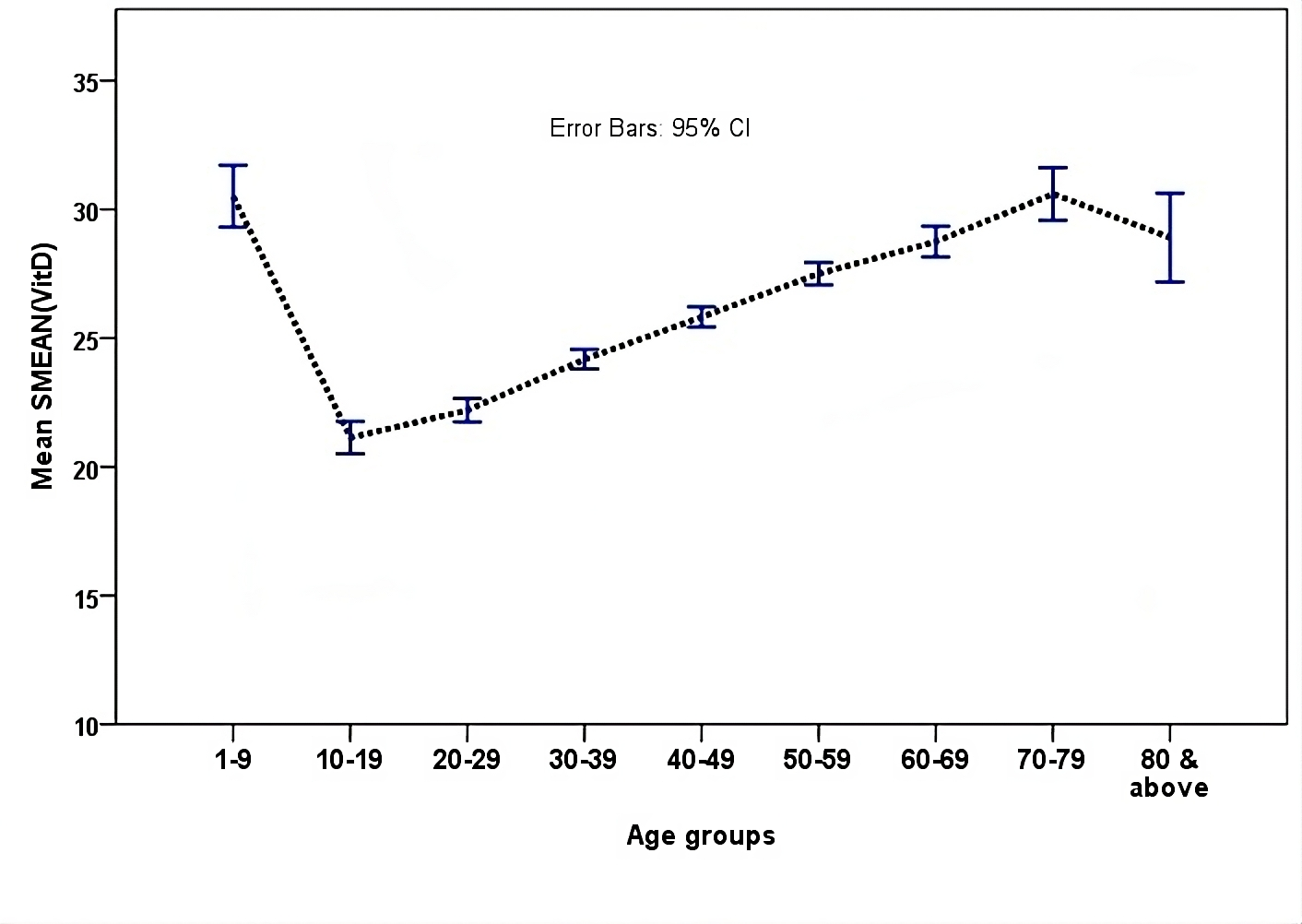


**Figure 3** Distribution of Vitamin D [25(OH)D] category among the age groups

**
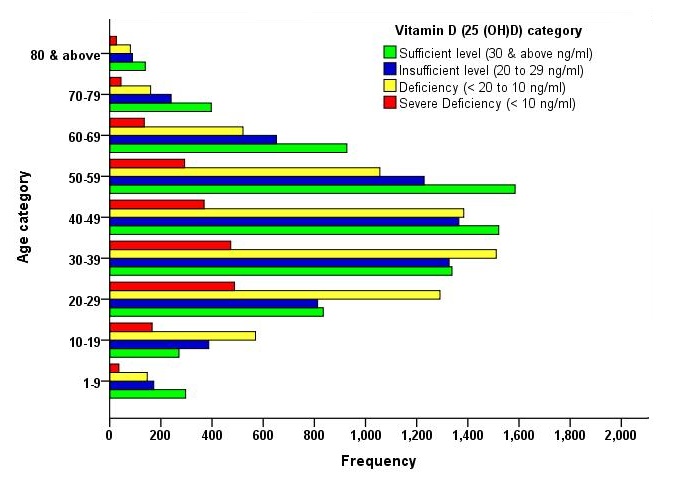
**

**Figure 4 Year-wise frequency distribution of severe deficiency (< 7 ng/ml) from the year 2017 to 2021 according to sex and age**


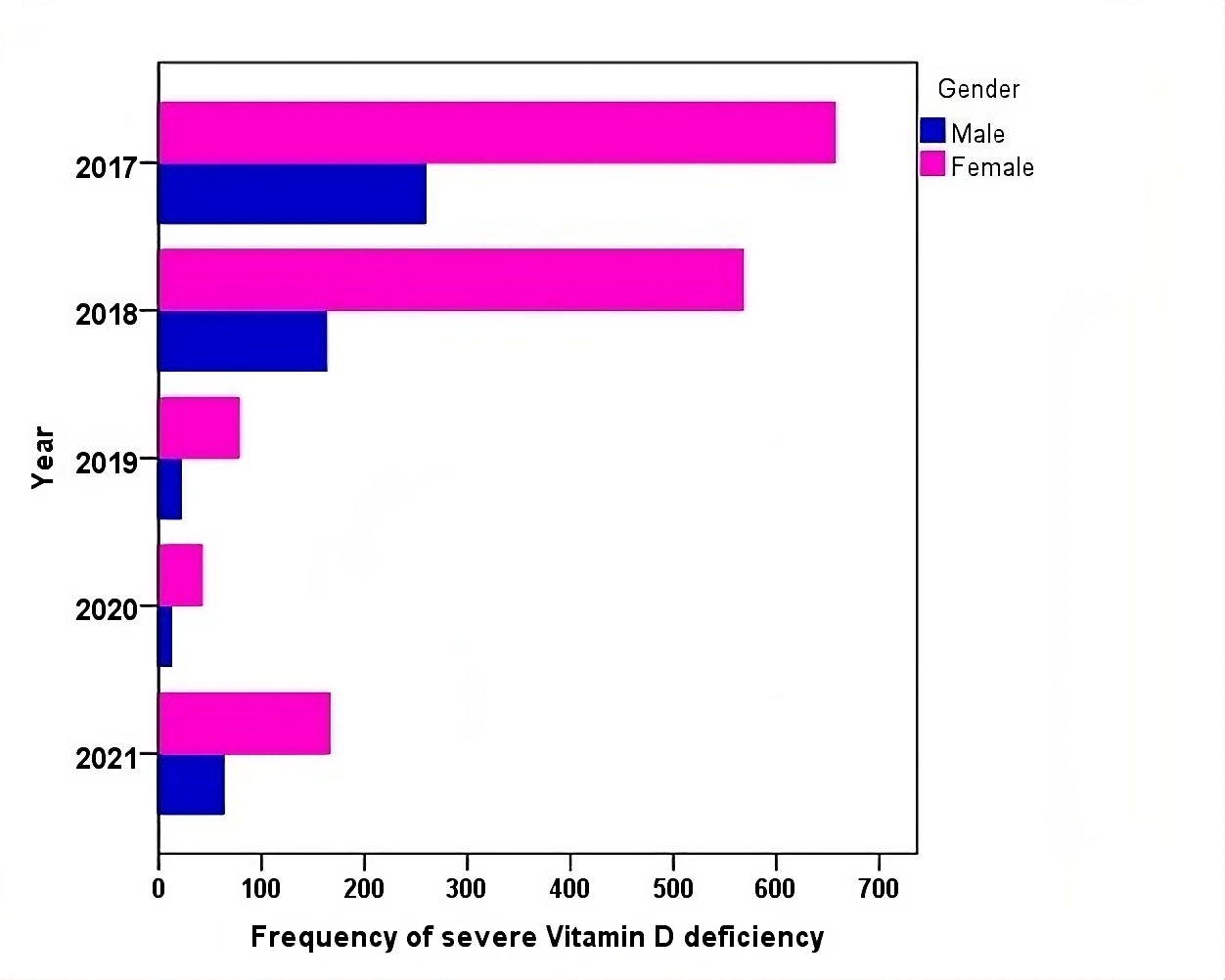

Supplement: Supplementary file 1 [file Table_1.DOCX]
